# Supplementary material for: An open-source framework for large-scale, flexible evaluation of biomedical text mining systems
Source: J Biomed Discov Collab. 2008 Jan 29;3:1. doi: 10.1186/1747-5333-3-1 (PMC2276192; doi:10.1186/1747-5333-3-1)
Supplement: Additional file 1 — Evaluation platform source code. The additional file contains the software evaluation framework discussed in this paper. The most current release can be downloaded from SourceForge.net [1]. See the accompanying README file for instructions on installation and use. Also included in the distribution is a collection of UIMA wrappers for some commonly used BioNLP tools and annotated corpora. [file 1747-5333-3-1-S1.zip › bionlp_uima_repository-1.0.1/docs/api/edu/uchsc/ccp/util/nlp/annotation/comparison/SloppySpanComparator.html]

SloppySpanComparator (Center for Computational Pharmacology Util/UIMA API)


|  |  |  |
| --- | --- | --- |
| **Overview** \| **Package** \| **Class** \| **Tree** \| **Deprecated** \| **Index** \| **Help** | |  |
| **PREV CLASS**   **NEXT CLASS** | **FRAMES**    **NO FRAMES**     **All Classes** |  |
| SUMMARY: NESTED \| FIELD \| CONSTR \| METHOD | DETAIL: FIELD \| CONSTR \| METHOD |  |


---


## edu.uchsc.ccp.util.nlp.annotation.comparison Class SloppySpanComparator

```
java.lang.Object
  edu.uchsc.ccp.util.nlp.annotation.comparison.SpanComparator
      edu.uchsc.ccp.util.nlp.annotation.comparison.SloppySpanComparator
```

**All Implemented Interfaces:**: Comparator<Span>

---

``` public class SloppySpanComparator extends SpanComparator ```

For "sloppy" comparison, the spans are required to overlap by at least one character. If the spans overlap, return 0, else return -1 is span1 occurs
prior to span2, 1 otherwise;

**Author:**
:   williamb

---

**Constructor Summary**

`SloppySpanComparator()`


| **Method Summary** | |
| --- | --- |
| `int` | `compare(List<Span> spanList1, List<Span> spanList2)`             Compare two span lists using the sloppy (overlap) match criteria. |
| `int` | `compare(Span span1, Span span2)`             Compare two spans using the sloppy (overlap) match criteria. |

**Methods inherited from class edu.uchsc.ccp.util.nlp.annotation.comparison.SpanComparator**

`matches, matches, overlaps, overlaps, sharesSpanEnd, sharesSpanEnd, sharesSpanStart, sharesSpanStart, sharesSpanStartOrEnd, sharesSpanStartOrEnd`

**Methods inherited from class java.lang.Object**

`clone, equals, finalize, getClass, hashCode, notify, notifyAll, toString, wait, wait, wait`

**Methods inherited from interface java.util.Comparator**

`equals`

**Constructor Detail**

### SloppySpanComparator

```
public SloppySpanComparator()
```


**Method Detail**

### compare

```
public int compare(Span span1,
                   Span span2)
```

:   Compare two spans using the sloppy (overlap) match criteria.

    :   **Specified by:**: `compare` in interface `Comparator<Span>` **Specified by:**: `compare` in class `SpanComparator`

---


### compare

```
public int compare(List<Span> spanList1,
                   List<Span> spanList2)
```

:   Compare two span lists using the sloppy (overlap) match criteria.

    :   **Specified by:**: `compare` in class `SpanComparator`
    :   **Parameters:**: `spanList1` -: `spanList2` - **Returns:**


---


|  |  |  |
| --- | --- | --- |
| **Overview** \| **Package** \| **Class** \| **Tree** \| **Deprecated** \| **Index** \| **Help** | |  |
| **PREV CLASS**   **NEXT CLASS** | **FRAMES**    **NO FRAMES**     **All Classes** |  |
| SUMMARY: NESTED \| FIELD \| CONSTR \| METHOD | DETAIL: FIELD \| CONSTR \| METHOD |  |


---
